# Fourier Transform Infrared Imaging—A Novel Approach to Monitor Bio Molecular Changes in Subacute Mild Traumatic Brain Injury

**DOI:** 10.3390/brainsci11070918

**Published:** 2021-07-12

**Authors:** Fazle Rakib, Khalid Al-Saad, Sebnem Garip Ustaoglu, Ehsan Ullah, Raghvendra Mall, Richard Thompson, Essam M. Abdelalim, Tariq Ahmed, Feride Severcan, Mohamed H. M. Ali

**Affiliations:** 1 Department of Chemistry and Earth Sciences, Qatar University, Doha P.O. Box 2713, Qatar; fazle@qsneoscience.com (F.R.); kalsaad@qu.edu.qa (K.A.-S.); 2 Department of Medical Biochemistry, Faculty of Medicine, Altinbas University, Bakirkoy, Istanbul 34218, Turkey; sebnem.garip@altinbas.edu.tr; 3 Qatar Computing Research Institute, Hamad Bin Khalifa University, Doha P.O. Box 34110, Qatar; eullah@hbku.edu.qa (E.U.); rmall@hbku.edu.qa (R.M.); 4 Qatar Biomedical Research Institute (QBRI), Hamad Bin Khalifa University (HBKU), Qatar Foundation (QF), Doha P.O. Box 34110, Qatar; ithompson@hbku.edu.qa (R.T.); taahmed@hbku.edu.qa (T.A.); 5 Diabetes Research Center, Qatar Biomedical Research Institute (QBRI), Hamad Bin Khalifa University (HBKU), Qatar Foundation (QF), Doha P.O. Box 34110, Qatar; emohamed@hbku.edu.qa or; 6 Department of Cytology and Histology, Faculty of Veterinary Medicine, Suez Canal University, Ismailia 41522, Egypt; 7 Department of Biophysics, Faculty of Medicine, Altinbas University, Bakirkoy, Istanbul 34218, Turkey

**Keywords:** fourier transform infrared, traumatic brain injury, biochemical analysis and immunohistochemistry

## Abstract

Traumatic brain injury (TBI) can be defined as a disorder in the function of the brain after a bump, blow, or jolt to the head, or penetrating head injury. Mild traumatic brain injury (mTBI) can cause devastating effects, such as the initiation of long-term neurodegeneration in brain tissue. In the current study, the effects of mTBI were investigated on rat brain regions; cortex (Co) and corpus callosum (CC) after 24 h (subacute trauma) by Fourier transform infrared (FTIR) imaging and immunohistochemistry (IHC). IHC studies showed the formation of amyloid-β (Aβ) plaques in the cortex brain region of mTBI rats. Moreover, staining of myelin basic protein presented the shearing of axons in CC region in the same group of animals. According to FTIR imaging results, total protein and lipid content significantly decreased in both Co and CC regions in mTBI group compared to the control. Due to this significant decrease in both lipid and protein content, remarkable consistency in lipid/protein band ratio in mTBI and control group, was observed. Significant decrease in methyl content and a significant increase in olefinic content were observed in Co and CC regions of mTBI rat brain tissues. Classification amongst distinguishable groups was performed using principal component analysis (PCA) and hierarchical clustering (HCA). This study established the prospective of FTIR imaging for assessing biochemical changes due to mTBI with high sensitivity, precision and high-resolution.

## 1. Introduction

Traumatic brain injury (TBI) is a complex neurological process with significant long-term neurobehavioral sequelae according to its severity [1,2]. The acute injury in the brain due to the external mechanical forces is the leading cause of traumatic brain injury which initiates immediate (at the time of acquaintance) disruption, shearing of axons (axons of neuron are torn due to stretch), hemorrhage and contusion [3]. Sub-acute and chronic injury caused by mitochondrial dysfunction, metabolic, cellular and molecular alterations evolve over hours to months with ultimate cell death (apoptotic and necrotic), inflammation and tissue damage at the end [2,3]. Although the damage in the brain can be focal, near the initial impact place, or diffuse and widespread, it is more common to see the latter one. Specifically extensive impairment of axons, i.e., diffuse axonal injury (DAI), is a very devastating pathology associated with TBI [4,5]. DAI is the shearing of axons when the brain shifts and rotates inside the skull resulting in damage in the axons [5,6]. Especially the junctions between the white (CC) and gray matter (Co) are most vulnerable in this condition [5,6,7].

In animal studies, mild to severe TBI is generated by merely altering the falling height of the weight. A falling height of 2 m using a weight of 450 g induces severe TBI (defined as 50% death in non-mechanically ventilated animals), whereas a falling height of 1 m induces mTBI (no mortality). Among the multiple modes to induce TBI, one of effective model is the Marmarou model [8,9]. A specific weight spontaneously falling through a Plexiglas tube from a certain height induces the trauma. The model has been well characterized histologically and animals with mTBI show clear DAI. Thus, the Marmarou model provides an ideal clinically relevant model to study the progression of brain damage, including DAI, in mild and severe TBI [8,9], which is used in this study.

A diffuse damage to the axons is believed to be the underlying pathology, but unfortunately, this is hard to diagnose or detect with conventional imaging modalities. Brain injuries are often diagnosed by using computerized tomography (CT) [10] and/or magnetic resonance imaging (MRI) [11,12]. To detect areas of bleeding (after a period of time remnant of blood called hemosiderin) CT and MRI imaging facilities are designed [13]. Enormous amount of red blood cells (RBC) must outflow from blood vessel/s to be detected by these imaging technologies. These technologies are unsuccessful in determining and discovering the existence of several, extensive and microscopic axonal injuries (that could have devastating neuropsychological deficits) unless multiple blood vessels are torn generating comparatively large bleeding [14]. Therefore, mapping/imaging methods, usoing Fourier transform infrared (FTIR) spectroscopy that provides complementary chemical information from injured brain, are sought.

Various functional groups of biomolecules, such as proteins, lipids, carbohydrates and nucleic acids, in samples can be analyzed based on their characteristic vibrational bands in FTIR spectra [15,16,17]. FTIR spectroscopy is considered as non-damaging, operator independent and timesaving technique for the characterization of different biological systems [18,19,20]. FTIR spectroscopy and imaging can be used for diagnostic purposes with body fluids [21,22,23], tissues [15,16,24,25,26,27,28] and cells [29,30,31,32,33,34,35] for biology and medicine [36,37,38,39,40]. Tragically, the mainstream mTBI brain injuries go undiagnosed. The non-appearance of the injuries on traditional imaging studies and the unintentional perception of an expected appearing and performing individuals are the two challenging factors to diagnose this injury [41]. In our previous study, the molecular changes in different brain regions due to stroke and mTBI 3 h after the injury were investigated by FTIR imaging [40,41,42]. In this paper, we aimed to investigate the imbalance in biochemical components in the cortex (Co) and corpus callosum (CC) of mTBI after 24 h from the injury by FTIR imaging for their greater implication on accuracy and sensitivity as a research tool.

## 2. Experimental Procedure

### 2.1. Ethical Statement

Animal supervision and all different experimental proprieties and procedures are executed very carefully with complete compatibility to the guidelines to use experimental animals by National Institute of Health (NIH). These experimental protocols also adhere to the rules of Netherlands Council on Animal Care and permitted by the prestigious University of Utrecht Animal Research Ethics Board (DEC 2013.I.08.063) prior to commencing any studies [16,41,43].

### 2.2. Animal Studies and mTBI Induction

A mild form of TBI were induced in Sprague Dawley male rats (Charles River Laboratories International, Wilmington, MA, USA), weighted 350–400 g, aged 11 weeks; by dropping a weight of 450 g from 1-m height inside a Plexiglas tube. This weight hits a stainless-steel disc that is glued (temporarily) to the skull. A foam bed supports the animal’s head. The fracturing of the skull is prevented by the steel disk and distributes the effect influence over a larger brain area. All rats were kept in a cage in groups of five at room temperature with a constant 12-h light/dark cycle.

Ten male rats (n_total_ = 10) were involved in the study and four rats were used as (age-matched) controls (n_control_ = 4) group. mTBI was induced in the right sensorimotor Co due to sudden hit. Twenty-four hours after mTBI induction, ten rats (four healthy control and six 24 h post TBI (n_mTBI_ = 6)) were anesthetized with isoflurane followed by transcranial perfusion and sacrificed by decapitation. The brains were extracted and kept at −80 °C until tissue sectioning and preparation for immunohistochemistry and FTIR studies [16,41,43].

### 2.3. Tissue Preparation for Immunohistochemistry and FTIR Imaging Study

After extraction, brain tissues 24-h post mTBI and control group rats were fixed in 4% paraformaldehyde (PFA) [16,41,43,44,45,46]. The brains were embedded in paraffin afterwards. At pragma −0.92 mm and using 10-μm thickness, paraffin-embedded brain blocks were serially cut into slices using a semi-automated rotational microtome Leica RM 2155 (Wetzlar, Germany). Hematoxylin and eosin (H&E), Luxol Fast Blue (Nissl) [46] and Hemosiderin [47] staining were performed to identify the affected morphology of the brain tissues that underwent mild injury. For immunohistochemistry, different primary antibodies, i.e., amyloid precursor protein (APP) [15,48], amyloid β (Aβ) [15,49], myelin basic protein (MBP) [15,50] and glial fibrillary acidic protein (GFAP) [15,30] tests were performed to identify protein accumulation and axonal shearing. The secondary antibodies were AlexaFluor 488, 568, and 647 and the nuclei were counterstained with 4′,6-diamidino-2-phenylindole (DAPI) stain as previously reported [51,52]. Further tissue preparation and detailed immunohistochemistry studies were performed as described in our previous studies [15,43]. Serial sections 10-μm in thickness were cut using a soft-tissue microtome and were used for FTIR imaging studies. The reason for using the adjacent cuts from each sample for staining and imaging studies was to identify the region of interests (ROIs) correctly in the Co and CC region in the brain tissues by comparing the H&E stained-sections with the FTIR images. Tissue sections were directly transferred onto IR transparent barium fluoride windows (BaF_2_) (6 mm thick) (Spectral Systems, New York, NY, USA). These four IR sections were kept in a desiccator with a vacuum pump in a cold room overnight to remove the moisture from the sections.

### 2.4. Fourier Transform Infrared Imaging (FTIR) Study

FTIR images were recorded using an Agilent FTIR Cary 620 micro-spectrometer (Agilent Technologies, Santa Clara, CA, USA) in the transmission mode at a spectral resolution of 4 cm^−1^ and spatial resolution 80 μm within the wavenumber range of 4000–700 cm^−1^ using a 64 × 64 μm mercury cadmium telluride (MCT) focal plane array (FPA) detector and there were 4 scan numbers per pixel. In each brain sample, three different areas from the cortex and corpus callosum regions were randomly chosen to collect IR maps by scanning the chosen areas, pixel by pixel (pixel size: 5.5 × 5.5 μm), and an IR spectrum was acquired from each pixel. A total of 7200 spectra were recorded from each chosen area (374 × 750 μm) of each section.

A FTIR micro-spectrometer (Agilent Cary 620, Cary, NC, USA) was used to record the FTIR spectra and maps within the range of 4000–700 cm^−1^ (spectral resolution 4 cm^−1^). More detailed instrument configurations and parameters used for this study were conducted as in our previous studies [15,16,25,41,42]. The FTIR spectra from two identical anatomical brain regions in the cortex and corpus callosum of different animals from each group were extracted from the FTIR images. The spectra were baseline corrected and averaged for data processing and analysis. The regions of interest (ROI) for spectral collection were determined by assessment of H&E-stained consecutive ROIs that were imaged in FTIR by mosaic imaging.

#### Pre-Processing and Analysis of FTIR Data

Agilent Resolution Pro. Software (version 5.0), Cytospec (version 2.00.06), MATLAB (version 2018a, USA) and OriginPro (version 2019) were used to analyze all spectral and imaging data. Area under the curve for of different bands of FTIR spectra (Table 1) was calculated to construct images/spectral maps of the functional groups to measure the different bio-chemical components in the mTBI and control groups.

Total protein is represented by the amide I/amide I + amide II band at 1700–1500 cm^−1^. The secondary structure of the protein band (Amide I) consists of α-helix structures at 1655 cm^−1^, a β-sheet at ~1645 cm^−1^ and a random coil at 1630 cm^−1^, as shown in Table 1 [15,17,30,53]. Total lipid was represented by the C–H stretching region—spectral range of 3000–2800 cm^−1^ which consists of asymmetric and symmetric CH_2_ (saturated lipid/lipid acyl) at 2915–2930 and 2860–2840 cm^−1^; CH_3_ asymmetric stretching ν_as_(CH_3_) (methyl concentration) at 2960–2950 cm^−1^; olefinic=CH at 3027–3000 cm^−1^ (unsaturated lipid) and lipid ester ν(C=O) (oxidative stress byproducts) at 1755–1715 cm^−1^. Lipid molecular and structural alterations, lipid/protein band ratios (that provide facts about the protein–lipid asymmetry and environmental changes, which lead to devastating effect on the cellular function), vector normalization, and second derivatives of raw spectra were used and calculated according to the details explained in our previous studies [15]. Relative quantification of the protein content and the distribution of amide I components were determined according to our previous studies [15,16,25,41,42]. The mean was generated for each animal group along with their standard deviation (significant level *p* < 0.0001). The MATLAB (curve fitting, predictive maintenance and bioinformatics) toolbox package was used to perform curve fitting for protein regions and other analyses in both experimental groups [15].

### 2.5. Chemometric Data Pre-Processing and Analysis

In the range of 4000–700 cm^−1^, principal component analysis (PCA) was performed on the collected IR spectra. The difference in the principal components helped to distinguish the brain groups. Unique fingerprint information about each group could be extracted from the PCA score and loading plots. The PCA score plots represent the degree of variability within the acquired spectra and were able to cluster the spectra of groups based on their chemical information. To distinguish the spectral types within the FTIR spectra of the brain (by shape preserving piece-wise cubic interpolation) from different experimental groups, unsupervised chemometric analyses, integrating principal component analysis (PCA) and hierarchical cluster analysis (HCA) were used. Hierarchical clustering analysis is an unsupervised learning technique which is used in conjunction with PCA to validate and visualize the results obtained via PCA. The detailed FTIR spectral composition and algorithmic calculation of the PCA [15] and HCA [15] correlations and variance analyses are described in our previous studies. The result was visualized in a dendrogram and the classification of control vs. mTBI brains can be obtained as images with color-coded clusters according to the heterogeneity scale [15].

### 2.6. Statistical Analysis

Statistical analyses were performed using GraphPad Prism (v8.1.2) and R (v3.6). FTIR results for each group (expressed as color-coded images and pixel population means) were compared using parametric Student’s *t*-test (with Welch’s correction) to highlight significant differences (after testing data for normal distribution and homogeneity of variance). Additionally, a one-way multivariate analysis of variance (MANOVA) was carried out on the six measurements using a Wilks test. The coefficient of variation was calculated for each parameter in each animal and the data were summarized as the mean and standard deviations for each group. The (two-tailed) *p* values less than or equal to 0.05 were considered statistically significant for multiple comparisons (* *p* ≤ 0.005; ** *p* ≤ 0.0001) with a 95% confidence interval.

## 3. Results

### 3.1. Histological Changes Post mTBI-Induction

**Figure 1:**
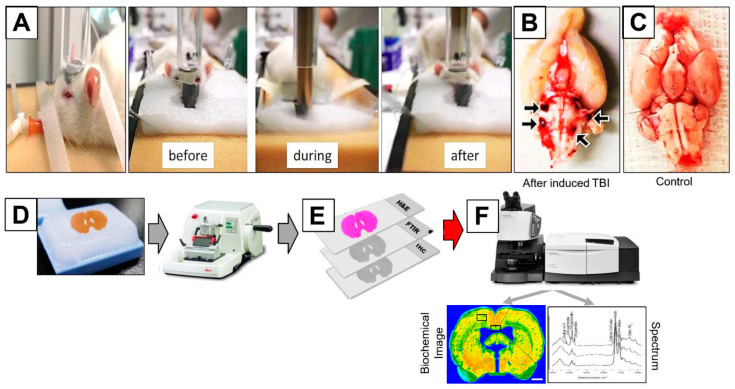
(**A**) Equipment and methods used to induce mTBI in rats. Displacement of an animal head as a result of the impact of a metal rod (weighing 450 g) dropped from a height of 1 m (the severity of TBI was controlled by changing the dropping height). (**B**) Brain showing hemorrhage in the stem and bleeding after mTBI. (**C**) Brain with no injury. Illustration of experimental workflow. (**D**) From the paraffin brain tissue block, 12 adjacent tissue sections of 10 μm in thickness were cut using a microtome. (**E**) The first two sections were H&E stained while the consecutive 4 sections remained unstained. (**F**) IR spectra were recorded from different region of interest (ROI) on the unstained sections and spectral data were processed and biochemical images of the brain tissue are obtained. The following 6 sections were tested for histology and elemental analysis.

As shown in Figure 1A, mTBI was applied successfully. The experimental rat brains were extracted, and the experimental brains experienced vascularity rapture and brain stem bleeding. This bleeding of the experimental brains was not shown in the control animals. The control and experimental rat brains were fixed, preserved in paraffin blocks, sectioned and then analyzed based on the work flow protocol mentioned in the experimental procedure section.

**Figure 2:**
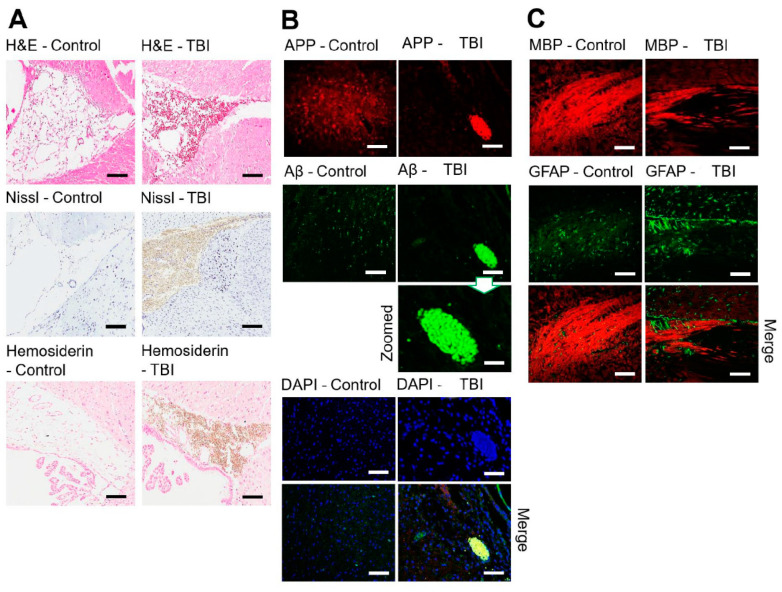
(**A**) Immunohistochemical comparison of the control and mTBI brain tissue areas (ROI) for H&E, Nissl and Hemosiderin staining. (**B**) Healthy control and injured brain sections labeled with APP (red), Aβ_1–42_ (green) and degenerated neurons with shrunken nuclei DAPI (blue) for the Co region. Aβ_1–42_ aggregation is shown. (**C**) CC region and disorganization of the myelin sheath (MBP, red) of the axonal neurons in the injured white matter are labeled with APP (red) and indicated activated astrocytes (GFAP, green stain). Scale bar = 100 µm and 20 µm (zoomed).

In the control, a normal morphology and uniform arrangement of neurons (with clearly visible nuclei stain) were observed in H&E and Nissl-stained sections under an optical microscope. Figure 2A shows the same cortical regions of the brain sections from both the control and experimental animals that were treated using the same staining procedure protocol. The H&E stain of the experimental brains identified swelling in the axons that were shown in the cortical region. This swelling might be due to accumulation of proteins and enzymes along the axons after mTBI axonal contusion. Axonal swelling causes axons to be separated from the neuron’s cell and can lead to neural degeneration. H&E staining images showed that there were cortical contusions, sub-cortical white matter, and CC damage associated with internal hemorrhages. Hemosiderin staining also confirmed blood/iron accumulation after injury, which clearly could not be identified in the control group. Figure 2A shows that the experimental brains, Luxol Fast Blue (Nissl) stained, experienced pallor compared to the control brain sections. The staining revealed not only a decrease in the general thickness of the recoloring for myelin but also organizational disorder in the white matter tissue.

### 3.2. Immunohistochemistry Studies on mTBI Brain Tissues

To gain comprehension of the properties of mTBI, affected and healthy areas of different brain regions were subjected to immunohistochemistry analyses using specific biomarkers (Figure 2B,C). Immunohistochemical analysis of sections established neuronal degeneration in the insulted brains with immunofluorescence studies emphasizing amyloid precursor protein expression. Comparing the control tissue sections with the injured brain tissue sections, we found APP and aggregated Aβ_1–42_ in the Co region (Figure 2B). These results imply that mTBI is not a simple unitary phenomenon, but rather is a more widespread, pernicious event that has implications for other functional brain areas [54]. MBP staining showed uniform axonal structure in the control group, while in the mTBI sections, the axons were sheared and displayed extensive axonal pathology (Figure 2C). GFAP and MBP were merged and clear astrocyte accumulation was observed near the damaged CC myelin sheath.

### 3.3. Bio Molecular Alterations in mTBI in Comparison to Control Group

#### 3.3.1. Qualitative Changes in Cortex (Co) vs. Corpus Callosum (CC)

**Figure 3:**
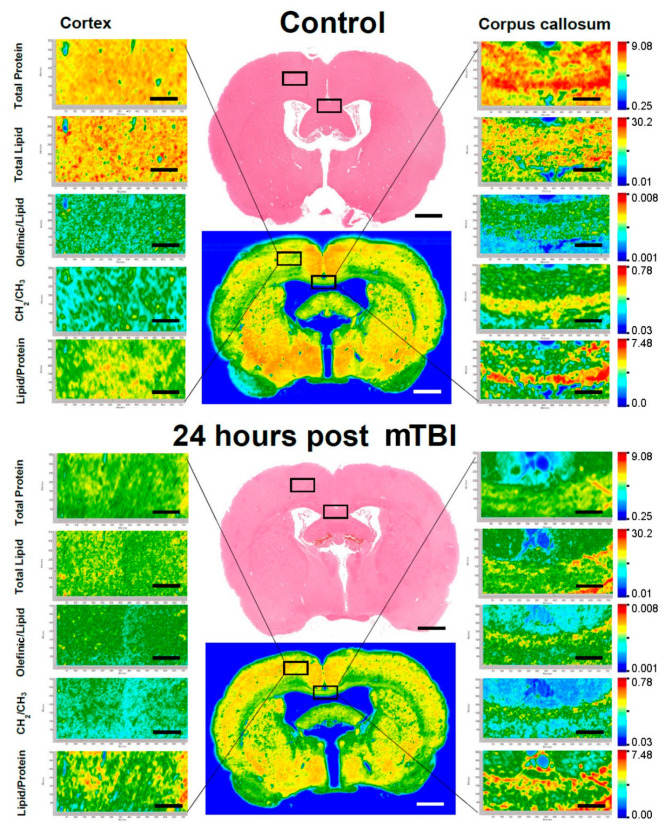
Representative FTIR images of the rat brain ROIs. Left-hand side showing Co region and right-hand side showing CC region. Top brain indicating the control group and the bottom brain indicating the affected group. Adjacent H&E image are placed to show the tissue morphology (Bregma −0.92 mm). Scale bar = 100 µm.

Low-resolution maps of whole control and injured brains were obtained at the beginning. Subsequently, the mapping areas were chosen and data from small sections were obtained using high-resolution pixel size (Figure 3). The FTIR images/spectral maps were generated in accordance with functional groups—total protein and total lipid content and olefinic/lipid, CH_2_/CH_3_ and lipid/protein ratios were calculated. These values were presented for the region of interest of Co and CC for both the control and 24 h post-mTBI groups. In Figure 3, the left-hand side represents the focused FTIR images of Co; thew right-hand side represents images from CC, extracted from the control (top) and 24 h post-mTBI (bottom) brains. Exact areas of analyses are marked with black squares for the Co and CC regions.

FTIR high-resolution imaging discriminated the Co (gray matter, which mainly consisted of proteins) region from the CC (white matter that has lipids as the main building block) regions based on the lipid and protein content. The Co FTIR maps showed that the mTBI FTIR images had low protein and lipid signals relative to the control maps. In addition, the FTIR maps of olefinic/lipids represented high concentration while CH_2_/CH_3_ represented low concentration in the experimental mTBI brains compared to the controls. In the CC of 24 h post-mTBI brains, there was a slight decrease in total protein concentration while the amount of total lipid also decreased moderately. These images were colored according to the calculated ratio values, where red represents the highest ratio and lowest ratio is presented by blue, as shown in the color bars.

#### 3.3.2. Quantitative Changes (Changes in Contents of Biomolecules) in Co vs. CC

In order to obtain quantitative information on the lipid peroxidation of the biological system, olefinic/lipid ratios were calculated. The olefinic/lipid ratio was calculated using the area ratio of olefinic band (=CH, unsaturated lipid) region over C–H stretching region [16,41,55,56]. The olefinic/lipid ratio represents an index of double bonds indicating the relative amount of unsaturated lipids in the lipid structure. Qualitative lipid acyl chain length changes were monitored by calculating area ratios of the CH_2_ antisymmetric band over the CH_3_ antisymmetric band region [16,41,55,56]. Additionally, total lipid and total protein amounts were obtained from CH_2_ symmetric/area of the C–H region and amide I/amide I + amide II ratios, respectively.

**Figure 4:**
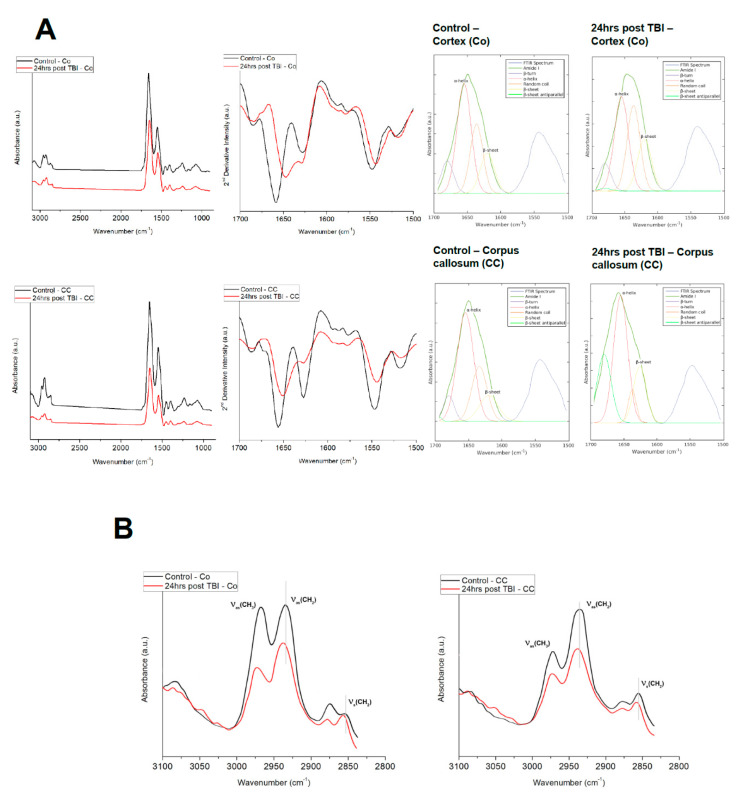
Representative FTIR averaged spectra. (**A**) FTIR averaged spectra in the range of 3100–700 cm^−1^ acquired from Co and CC region of control gray matter and white matter. Average second-derivative spectra of the amide I band (spectral range of 1700–1600 cm^−1^). The spectra show α-helical (secondary protein) structure at 1655 cm^−1^ and β-sheets protein conformation at 1630 cm^−1^. Representative curve fitting of the amide I band in the spectral range of 1700–1600 cm^−1^ comparing the control and 24 h post-injured Co and CC. (**B**) Representative FTIR spectra averaged from control (black) and mTBI (red) for lipid region in Co and CC.

The FTIR spectra (Figure 4A) revealed the distribution of total protein in the injured brain; where total protein and total lipid content levels in mTBI were significantly reduced compared to the control brains in the cortical hemisphere. Moreover, in both regions, lipid to protein ratio was significantly increased in the mTBI groups compared to the control. Comparing the ratio values for CH_2_/CH_3_ and olefinic/lipid, the values showed a decrease in the CH_2_/CH_3_ area ratio, but an increase in the olefinic/lipid area ratio in comparison to the control brain Co (Table 2). While analyzing the ratio values for the CC, it is interesting to note that, though the total protein and total lipid values decreased in mTBI group, significant variations were observed in the lipid/protein ratio between these two groups. Moreover, CH_2_/lipid values declined and olefinic/lipid ratio values showed an increase in the affected brain CC compared to the unaffected or healthy rat brain regions.

#### 3.3.3. Protein Secondary Structural Changes in Co vs. CC

FTIR spectra were collected from Co and CC regions (from various locations) of control and 24 h post mTBI brains in order to quantify IR absorption for protein components (Figure 4A). These overlaid spectra were baselined and normalized. The average FTIR spectrum for controls is denoted by the black and red line and represents mTBI spectrum. The average IR spectra of Co and CC for both groups were dominated by amide I absorption in the range of 1600–1700 cm^−1^ with a moderate absorption of amide II band at 1560–1590 cm^−1^. The spectra collected from different points/pixels within the Co region of the control brains showed a high similarity in terms of position, amplitudes and line shape. The second derivative spectra showed that the amide I band (protein secondary structure) is the main contributor and was centered between 1645–1655 cm^−1^, which arises from α-helical conformations. The amide I band was composed of α-helix at 1655 cm^−1^, parallel native β-sheet proteins ≈1630 cm^−1^ and a random coil at ~1630–1640 cm^−1^.

The variations in the estimated secondary structure values based on the amide I bands are shown in Table 3. In both Co and CC, α-helical composition decreased from 49.38% (control) to 24.01% (mTBI); β-sheet increased from 32.41% (control) to 73.14% (mTBI) and random coil was 19.13% for control and 3.22% for mTBI. The results indicated that the protein in both Co and CC of the control group retained its native α-helical structure but there were significant changes in the mTBI where the α-helical to β-sheet transformation took place. 

#### 3.3.4. Physical Structural (Lipid Order and Lipid Acyl Chain Flexibility) Changes in Co vs. CC

Figure 4B shows the spectral changes in the lipid region 2800–3100 cm^−1^ that can give an indication about lipid order and lipid acyl chain flexibility for Co and CC from control and 24 h post-mTBI groups. The lipid order was investigated by analyzing the alterations or shifts in the wavenumbers of ν_s_CH_2_ and ν_as_CH_2_. It is evident from the figure that, in Co, the lipid acyl spectral bands ν_s_CH_2_ and ν_as_CH_2_ at 2852 and 2925 cm^−1^, respectively, shifted to higher frequency values, 2853 and 2927 cm^−1^, accordingly in the mTBI Co than in those of the control samples. Similarly, while analyzing mTBI CC, the wavenumbers associated with lipid, a significant shift towards higher frequencies were also calculated which are 2854 and 2928 cm^−1^ for ν_s_CH_2_ and ν_as_CH_2_ accordingly. This increase in the frequency values of the lipid bands indicates a decrease in lipid order, e.g., increase lipid disorder and an increase in acyl chain flexibility [37,45,46] Furthermore, the lipid membrane fluidity was investigated by analyzing the alterations in the bandwidth of ν_as_CH_2_. The bandwidth values of this band were higher in Co (2.31 ± 0.002, *p* < 0.001) and in CC (2.57 ± 0.001, *p* < 0.001) of mTBI in comparison to the control group Co (1.7 ± 0.002) and CC (1.8 ± 0.001), respectively. The significant increase in the band width indicates an increase in lipid dynamics, e.g., membrane fluidity in the (mTBI) injured animal group (Figure 4B) [45,46]. 

### 3.4. Principal Component Analysis (PCA)

**Figure 5:**
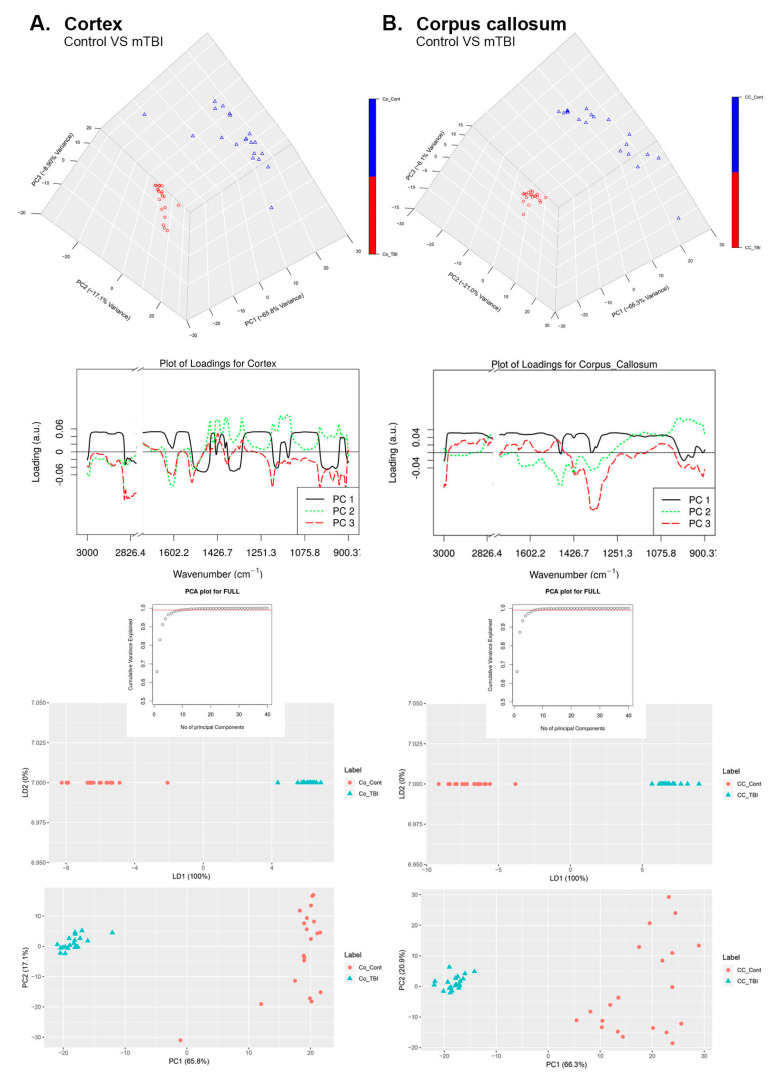
Principal component analysis of control and mTBI groups. Supervised linear discriminant analysis (LDA) is presented besides relative loading plots and the score plots are presented to emphasize the significant differences in the data collected from control and mTBI FTIR spectra in (**A**) Co and (**B**) CC.

PCA (Figure 5A,B) was performed on the spectral second derivatives in the range of 4000–700 cm^−1^ (range of 2500–2000 cm^−1^ was removed) for the control and mTBI brains from Co (Figure 5A) and CC (Figure 5B). Loading plots and PCA plots were also given, along with their 3D PCA diagrams. Spectral differences were clearly seen from the loading plots between the groups. The distinctions were principally in the range of 2525–2980 cm^−1^, which associated to the molecular changes in the lipids. Moreover, there were molecular variations in the spectral region of 1530–1680 cm^−1^, which was identified by proteins in the brain tissue. The scores plot uncovered that spectral information gathered from the rat cerebrum cortices were clustered into two particular gatherings that associated with control and influenced by mTBI. The unmistakable isolation between the two gatherings in the PCA score plots demonstrated that the molecular makeup of the cortical brain tissue changed because of mTBI with the primary 3PCs representing ~66% of the total variation. Another crucial ~17–21% were impacted by various factors. 

For the Co region of the brain, we observed from Figure 5A, that PC1 captures 65.8% of the total variance in the data while PC2 and PC3 capture 17.1% and 8.90% of the total variance in the spectral collection, respectively. The loading vector for PC1 (Figure 5A) was predominantly positively correlated with all wavelengths. Moreover, PC1 can easily differentiate the control samples (positive values in PC1) from injured brain samples (negative values in PC1). The loading vector of PC2 was predominantly negatively correlated with the wavelengths of the 3000–1450 cm^−1^ region, whereas it was primarily positively correlated with the wavelengths of the 1450–900 cm^−1^ region. Finally, the loading of PC3 was mostly negatively correlated with all wavelengths and captured the maximum variation between the regions of 2980–2525 cm^−1^, which corresponded to the lipid bio molecular changes.

For the CC region of the brain, we observed from Figure 5B that PC1 captures 66.3% of the total variance while PC2 and PC3 capture 21.0% and 6.1% of the total variance in the spectral collection, respectively. The loading vector of PC1 (Figure 5B) was positively correlated with all wavelengths. Moreover, PC1 can easily differentiate the control samples (positive value in PC1) from the injured brain samples (negative values in PC1). Similarly, the loading vector of PC2 was negatively correlated to wavelengths in the range of 2000–1250 cm^−1^ and was positively correlated to the remaining wavelengths. PC2 also helped to distinguish control samples (negative values in PC2) from the mTBI samples (positive values in PC2). Finally, the loading vector of PC3 was maximally negatively correlated to wavelengths in the 1400–1300 cm^−1^ region, which was related to the proteins in the brain. Interpreting the dendrogram analyses, we can conclude that all the regions that have a high content of lipids are clustered together, based on their lipid bio-chemical make-up.

Supervised linear discriminant analysis (LDA) was performed on top of the results obtained from the PCA model (three principal components). With LDA, we could observe that the control samples (CC) could be differentiated from stroke samples (CC) along the 1st LDA dimension (LDA1 100% in plot). Similarly, we could observe that the control samples (cortex) could easily be segregated from the stroke samples (cortex) along the 1st LDA dimension (LDA1 100% in plot).

### 3.5. Hierarchical Clustering Analysis (HCA)

**Figure 6:**
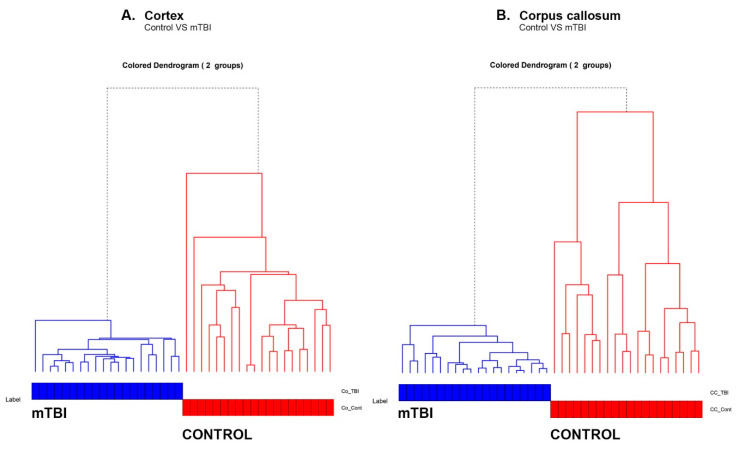
Hierarchy cluster analysis (HCA) showing the heterogeneity of the spectra belonging to the control and mTBI groups due to the bimolecular changes in (**A**) Co and (**B**) CC regions.

This separation between the control and mTBI Co and CC can be clearly seen in the hierarchical dendrogram (Figure 6A,B). We generated a hierarchical dendrogram for all the spectral samples belonging to the control and injured rat brains. We then generated a dendrogram using the “A2Rplot” function from the A2R package in R. The resulting dendrograms are shown in Figure 6A,B for Co and CC experiments, respectively. We can observe that all the samples belonging to the control formed part of one cluster whereas the injured rat brain spectral samples formed a different cluster.

## 4. Discussion

Though mTBI is a well-known neurogenerative disease, the biochemical and bio-molecular changes in the brain due to mild effects remain undiagnosed. Brain tissue is composed of different sensitive regions, and Co and CC are the most vulnerable ones according to previous studies [7,41]. In the present study, detailed FTIR spectroscopic analyses revealed that the Co/gray matter spectra were quite different from the CC/white matter spectra (especially for lipid vibrational bands). They have two different densities since Co/gray matter comprises cell bodies and CC/white matter comprises axons [41,57]. The shearing force (soft fragile brain scrapping against the hard and jagged inner surfaces, also known as flax, of the skull), which is the eventual upshot of mTBI, creates axonal injuries. In these axonal injuries, the axon can be completely or partially torn This incident is called axonal shearing or diffused axonal injury [5,58]. 

If some of the larger and resilient arteries are also torn, there is no bleeding (hemorrhage) in the brain. Thus, CT or MRI traditional imaging techniques, which are designed to detect bleeding [13], are not sensitive enough to detect individual axonal injuries. In the current study, to reveal a complete anatomic and biochemical picture of mTBI after 24 h of induction compared to a control group, we evaluated how a spectroscopic imaging platform can be combined with routine histology and immunohistochemistry, revealing a wealth of biochemical information in addition to anatomic information close to the cellular level which is an asset of our spectroscopic imaging approach [15,39,40,41,42].

The spectral information, including the frequencies, bandwidths, and peak areas of the vibrational modes of biomolecules for the two groups at different regions of interest (Co and CC), is quite helpful to monitor variations in the structure and content of different biomolecules, such as proteins, saturated and unsaturated lipids, nucleic acids, etc. Due to the inhomogeneity of the brain tissue, it is difficult to compare two different regions of the same tissue. The current study focused on comparing the biochemical changes in the same regions of control and injured brains. 

### 4.1. Control—Co vs. mTBI—Co

The C=O stretch which is the origin of the amide I band has little commitments from the N—H twisting of proteins. This band is used to assess total content and conformation of proteins in neurogenerative sicknesses. Alterations in proteins reflect certain distinctions in its secondary structure, which includes α-helix, β-sheet, and random coil [15,41]. Accumulation of Aβ was observed in damaged Co/gray matter of 24 h post-mTBI animals. By comparing immunohistochemical images with the corresponding FTIR maps, it can be concluded that the distribution of the amide I band in the FTIR maps corresponded strongly to the Aβ-positive area of Co, in mTBI, in comparison to the control group. Degenerated neurons with shrunken nuclei (DAPI—nuclear stain, blue) were also observed in the injured tissue from Co and the merging of APP, Aβ and DAPI. A statistically significant difference was seen in the absorption value of protein bands in this area, as shown in Table 2. This study proposes that α-helix structures are major contributors to the control gray matter while β-structures are prominent in mTBI due to the denaturation of axonal cytoskeleton and exoplasmic proteins deposits in β-structures [59].

In a previous study [60], the brain Aβ deposits and levels of isoprostanes generated by lipid peroxidation in the brain and urine were investigated in a Alzheimer disease model mouse with TBI. It is reported that there is a link with TBI and the mechanism of AD by showing that TBI accelerates brain Aβ accumulation and oxidative stress, which they suggest could work synergistically to promote the onset of or drive the progression of AD.

The primary injury in TBI is followed by secondary injury in the hours or even days after the traumatic event, which leads to biochemical, cellular, and physiological changes, including inflammation, mitochondrial dysfunction, and the generation of oxidative stress. Oxidative stress further leads to lipid peroxidation and oxidation of proteins and DNA [61]. Aldehydes and carbonyl compounds are the degradation products of peroxidation of lipids, causing smaller fragments by breaking down longer chains [15,41,62]. Additionally, information about lipid peroxidation can be determined by calculating olefinic=CH/lipid ratio in FTIR studies [21,33,63]. In our study, based on the significant increase in olefinic/lipid ratio and also in lipid/protein ratio in the experimental mTBI animal group compared to the control, an increase in lipid peroxidation products, such as malonaldehyde and 4-hydroxynonenal, was indicated in Co region of the mTBI group [63] suggesting a biomarker panel for mTBI diagnosis, which also includes the measurement of malondialdehyde-modified low density lipoprotein (MDA-LDL) due to the elevation of malondialdehyde level in mTBI patients.

Lipid order state was inferred from the shifts in the frequency of the CH_2_ symmetric and asymmetric stretching bands. In the system, a higher frequency corresponded to a higher lipid disorder—(escalation in acyl chain flexibility) [41,58,59]. Alterations in ion channels and receptors are ultimately due to changes in the lipid content of the membrane and the physical property of membrane asymmetry and thickness [60]. Thus, functional disorders in brain tissue are associated with alterations observed in lipid structure in the membrane [41]. Although information about the TBI-mediated diffuse neuronal membrane disruption is limited, it was shown that membrane disruption has been shown to occur acutely following injury, primarily within neurons [64]. In a current study, membrane disruption was investigated over a longer temporal profile, from 6 h to 4 w, following diffuse TBI induced using the central fluid percussion injury (CFPI) model in rats. It was implied that membrane disruption displayed a biphasic pattern, where nearly half of the neurons were membrane-disrupted sub-acutely, from 6 h to 3 d post-TBI [64].

In the current study, the membrane fluidity in both Co and CC brain regions was shown to be increased in mTBI animal groups compared to the control group. Consistent with our finding [41], it was suggested that neuronal excitability was a significant component of the secondary injury cascade that accompanies TBI, and can be correlated with an alteration in the lipid/protein oxidation, membrane fluidity, and Na(+), K(+)-ATPase activity. All these changes can be caused by the oxidative stress that accompanies TBI [65,66].

### 4.2. Control—CC vs. mTBI—CC

CC is the most critical and delicate structure, consisting of axons, and allows communication between two opposite sides of the brain. During mTBI, CC can be badly damage by colliding with the rigid Flax Major differences were clearly found between the 24 h post-mTBI and control groups. GFAP acted as an astrocyte marker. Disorganization of the myelin sheath was identified by MBP. In the post-mTBI group, the thickness/density of the myelin sheath decreased with MBP broke down. This increases the vulnerability of exposed axons as well as behavioral deficits and functional connectivity tend to decline [15,67]. MBP dysfunction(s) are related with physiological reactions to stress and emotional states, nervousness, and depression in adulthood and every conceivable marker for rationalities in cerebrum injuries are accounted for in other studies [15,43]. Twenty-four hours post closed head injury seems to be more serious and consistent with histological examination due to our observation of the spectral patterns in axonal injury of CC. Significant biochemical changes in lipids and proteins are accompanied by this effect. In terms of lipids, the 24-h group had greater concentration at 2925 and 2852 cm^−1^, but a lower one at 2955 cm^−1^. There were significant changes in unsaturated lipids, as well as a revealed lesser concentration at 3012 cm^−1^. These findings demonstrate that multiple chemical components in the CC of rats can be discriminated by highly sensitive FTIR spectroscopy. The outcomes from FTIR imaging uncovered that, in spite of the fact that the relative aggregated protein substance is expanded inside the cortical locale, there was not a huge increase in the collected protein substance near the CC. Additionally, although there was a net reduction in protein concentration inside the CC, there was no change in the lipid to protein ratio. This suggests that these molecular alterations are not a consequence of chemical changes, which would almost certainly modify the lipid to protein ratio as referenced previously. Analyzing both findings of molecular changes 3 h post mTBI [41] and our recent 24 h post-mTBI study, it is evident that in both time points—(a) there is a reduction in protein content; (b) biochemical alterations affected the secondary structure of protein; (c) reduction in total lipid content is also observed; (d) unsaturated lipid content is increased in both regions of the brain; and (e) the uniformity of lipid/protein ratio data indicated that in mTBI animals there is no chemical effect responsible for these molecular changes.

## 5. Conclusions

Due to high sensitivity, the present study reflected on the potential of FTIR imaging in detecting temporal biochemical changes in 24 h post mTBI rats. The study also demonstrates a distinct bio-molecular gradient with changeovers between healthy and injured tissues, allowing identification and delineation of the affected areas. The uniformity of the lipid/protein ratio data indicated that oxidative damage or chemical effects are not responsible for the observed molecular alterations within the Co and CC. These results could not be obtained using traditional MRI or CT alone, as these techniques lack resolution and sensitivity to cellular levels. We speculated that therapeutic intervention would be beneficial if focusing on minimizing axonal damage. Since we observed molecular alterations within these critical regions at this time point, results may be reversible and lack of chemical modification to protein and lipid is detected within the Co and CC at 24 h after the mild traumatic injury. Using multivariable analyses, which included PCA and HCA, different time points were successfully classified based on the acquired spectral fingerprints. The chemical components related to proteins and lipids greatly contributed to this distinction. Our study paves the way for FTIR spectroscopy with multivariable analyses to estimate the accurate biochemical alterations due to chemical and mechanical effects post injury at a network level. To elucidate chemical changes, further work should be designed with more time points, more experimental animals, and including additional regions of interests, which are vulnerable to injury.

## Figures and Tables

**Table 1:** Spectral regions used for analytical interpretation for mTBI study [15,16,25,41,42].

	Infrared Band Assignment	Spectral Range (cm^−1^)	Comments
**Protein components**	Amide I	1700–1600	Proteins (80% C=O stretching, 10% N–H bending, 10% C–N stretching) [50]. Specifically sensitive to protein secondary structure
	Amide II	1555–1535	Proteins (60% N–H bending, 40% C–N stretching) [50]
	β-sheet	~1630	Amide I—β-sheet protein secondary structure
	α-helix	1655–1645	Amide I—α-helix protein secondary structure
	Random coil	1645–1630	Amide I—protein secondary structure
**Lipid components**	CH_2_ symmetric stretching	2852–2800	Mainly associated with lipids
	CH_2_ asymmetric stretching	2915–2930	Mainly associated with lipids
	CH_3_ asymmetric stretching	2950–2960	Mainly associated with lipids and protein side chains
	C–H stretching	2994–2800	Total lipid region
	Olefinic=CH	3000–3027	Unsaturated lipids

Ν = stretching vibration; ν_as_ = asymmetric stretch; ν_s_ = symmetric stretch.

**Table 2:** The changes in content and area ratios of some biomolecules in control and mTBI groups.

	Control Co	mTBI 24 h Co	Control CC	mTBI 24 h CC
Total protein	5.8 ± 0.047	4.42 ± 0.221 **	4.2 ± 0.211	3.92 ± 0.019 **
Total lipid	19.72 ± 0.098	15.07 ± 0.075 **	25.43 ± 0.171	23.77 ± 0.119 **
Olefinic/Lipid	0.0043 ± 0.004	0.4204 ± 0.028 **	0.0027 ± 0.002	0.0048 ± 0.003 **
CH_2_/CH_3_	0.227 ± 0.019	0.205 ± 0.015 **	0.321 ± 0.028	0.226 ± 0.066 **
Lipid/Protein	3.397 ± 0.170	3.408 ± 0.017 *	6.017 ± 0.302	6.062 ± 0.036 *

The values are shown as mean ± standard deviation for each group. *n* = 10 and the degree of significance was denoted as: * *p* < 0.005, ** *p* < 0.0001 and obtained by comparing each treated group with the control group. *Co*=*Co* and *CC*=*CC.*

**Table 3:** Relative amounts of the secondary structural protein contents were quantified from the curve fitting of original absorbance spectra of amide I band.

	Control Co	mTBI 24 h Co	Control CC	mTBI 24 h CC
α helix	0.49 ± 0.038	0.24 ± 0.011 **	0.46 ± 0.022	0.30 ± 0.031 **
β sheet	0.32 ± 0.041	0.73 ± 0.014 **	0.40 ± 0.041	0.62 ± 0.024 **
Random coil	0.19 ± 0.013	0.03 ± 0.022 *	0.14 ± 0.0071	0.08 ± 0.032 *

The values are shown as mean ± standard deviation. The degree of significance was denoted as: * *p* < 0.05, ** *p* < 0.0001.
